# Nutritional intakes and associated factors among tuberculosis patients: a cross-sectional study in China

**DOI:** 10.1186/s12879-019-4481-6

**Published:** 2019-10-29

**Authors:** Zhewen Ren, Fei Zhao, Hui Chen, Dongmei Hu, Wentao Yu, Xiaoli Xu, Dingwen Lin, Fuyi Luo, Yueling Fan, Haijun Wang, Jun Cheng, Liyun Zhao

**Affiliations:** 1 National Center for Tuberculosis Control and Prevention, Chinese Center for Disease Control and Prevention, Beijing, China; 2 Clinical Trial And Research Center, Beijing Hospital, Beijing, China; 3 National Institute for Nutrition and Health, Chinese Center for Disease Control and Prevention, Beijing, China; 4 Guangxi Zhuang Autonomous Region Center for Disease Control and Prevention, Nanning, China; 5 Linyun County Center for Disease Prevention and Control, Baise, China; 6 Shanxi Provincial Center for Disease Control and Prevention, Taiyuan, China; 7 Lin County People’s Hospital, Lvliang, China

**Keywords:** Tuberculosis patients, Nutrient intakes, Socio-demographic factors, Behavioral factors

## Abstract

**Background:**

The objectives of this study were to examine nutrient intakes of tuberculosis (TB) patients and to identify their associated factors.

**Methods:**

In this cross-sectional study, 300 adult TB patients were surveyed in two impoverished counties in China. Nutrient intakes were evaluated through two consecutive 24-h dietary recalls and compared with the Chinese Dietary Reference Intakes (DRIs) 2013. The potential socio-demographic and behavioral factors were analyzed using multivariate logistic model to identify strong influential factors.

**Results:**

We found that mean daily energy intake was 1655.0 kcal (SD: 619.3 kcal) and 1360.3 kcal (SD: 552.1 kcal) for male and female patients, respectively. The mean daily energy intake was significantly lower than that has been recommended by DRI (i.e., 2250 and 1800 kcal for males and females, respectively), with 87.4% of the male patients and 59.9% of female patients failed to consume adequate energy. The protein intakes were 44.6 g (SD: 18.2 g) and 35.9 g (SD: 12.3 g) for male and female patients, respectively, which were lower than the recommended values by DRI (i.e., 65 and 55 g for males and females, respectively). Most male (90.8%) and female (58.4%) TB patients had insufficient daily protein intake. Further analyses suggested that mean daily intakes of many micronutrients, were insufficient, while for most of patients, intakes of vitamin E and sodium were sufficient. We identified that unemployment was a risk factor for low energy intake (*p* < 0.05) and out-home-eating was a protective factor for low protein intake (*p* < 0.01).

**Conclusions:**

In impoverished areas in China, intakes of macronutrients and most micronutrients in TB patients were inadequate compared with DRIs, especially for unemployed patients and patients eating at home. These findings suggested that public health actions are needed to promote education on TB patients about significance of nutritional support, and, further interventions in TB patients’ nutritional intakes are also required.

## Background

Tuberculosis (TB) is an airborne disease caused by *Mycobacterium tuberculosis* and is the leading cause of death from single infectious agents. According to World Health Organization (WHO), there were estimated 10.0 million people developed TB disease and 1.6 million people died from TB in 2017. China is in 2nd place among 30 TB high burden countries, with a reported incidence of 63 per 100,000 persons per year in 2017 [1].

The interactions between tuberculosis and malnutrition are well appreciated. TB can cause weight loss or cachexia and malnutrition can predispose to TB. On one hand, TB patient requires more energy to maintain body function that caused by increasing basal metabolic rate (BMR), leading to weight loss [2]. On the other hand, food intake may be negatively affected for TB patients due to reduction in appetite and gastrointestinal disorder [3], resulting in undernutrition. Malnutrition can further lead to impaired immune function [4, 5], as nutritional deficiency alters the interaction between macrophages and T-lymphocytes [6]. Moreover, although most people who get infected from TB will not manifest symptoms as their immune system manages to control the bacteria, malnourished persons are more likely to develop active TB because the infection is no longer constrained by their immune systems [7].

Assessment of nutrient intakes is critical in nutritional management of patients with TB. Enough nutrient intakes is essential for combating TB. Insufficient protein and caloric intakes can hinder the functions of some generalized host defense mechanisms that are essential for combating TB [8]. Additionally, both vitamins and minerals play important roles in immunity [9], deficiency of one or more of these nutrients will impair person’s resistance to any infection [10]. Although previous studies in China suggested that protein, caloric and micronutrients intakes were inadequate in TB patients [11–13], few studies has been conducted to explore potential drivers. In this study, we 1) examined whether nutrient intakes for TB patients was sufficient in two impoverished counties in China; 2) identified the potential factors that caused the insufficient consumption of energy and protein.

## Methods

### Study context

This cross-sectional study was undertaken as part of the project “Investigation of nutrition and diet of patients with pulmonary tuberculosis in poor areas in China” supported by the World Health Organization Regional Office in the Western Pacific. It was conducted from November 2015 to April 2017 in two counties, Lingyun county in Guangxi province and Lin county in Shanxi Province. These two counties were national impoverished counties, and did good job in TB management. TB case notification rates in 2016 were 106.97 per 100,000 and 63.16 per 100,000 in Lingyun county and Lin county, respectively.

### Participants

We focused our study on adult patients (age ≥ 18 years) with active TB who were registered in *Tuberculosis management information system* from Nov 1st, 2015 through May11^th^, 2017. Patients with extrapulmonary tuberculosis, and those aged 18 years and below or with severe complications were not eligible for the study. Pregnant or breast-feeding women, and those who declined to sign the consent form were also excluded.

### Sample size

As BMI is commonly used in nutrition assessment, we applied the prevalence of BMI < 18.5 which is also defined as malnutrition [14] in determining sample size. We assumed that the prevalence of malnutrition in the general population in poor areas and TB patients would be 6.7% [15] and 25.0% [2], respectively, and the required sample size was calculated to detect difference in the two proportions. The probability of a type I error was set at 0.05, the power of the study was estimated at 90% and the design effect was set at 1, determining a sample size of TB patients was 77 per study site. Considering participants’ refusal, we expanded the sample size to 150 per study site, and the final sample size of TB patients was 300 totally.

### Socio-demographic and behavioral factors

Data for associated factors were taken from a questionnaire, including socio-demographic attributes (age, gender, education level, marital status, occupation and household income level) and behavioral variables (alcohol consumption, smoking status and eating out-of-home).

In line with Chinese Dietary Reference Intakes (DRIs) 2013 [16], we categorized patients’ ages into 3 groups: 18–49 years, 50–64 years, 65 years and above to facilitate comparisons. Educational level was classified into primary school and below, junior middle school and above. Household income level was proxied as annual household income and was grouped into < ¥20,000, ¥20,000 - ¥40,000, and ≥ ¥40,000 [17]. Alcohol consumption was surveyed as drinking wine, beer and Chinese spirit now or ever. A smoker was defined as smoking now or smoking previously but have stopped smoking in the evaluation period. Eating out-of-home was defined as eating at least one meal away from one’s own home or their residents’ home during the survey [18]. Severity of TB was grouped into two categories based on chest x-ray. Specifically, if the lesion was confined to two lung fields then it was defined as mild. If the lesion covered more than two lung fields or there were cavities, it was defined as severe [19].

### Assessment of nutrient intake

Trained staffs performed face-to-face interviews for each participant to obtain dietary intake data through a 2-day 24-h dietary recall (24hdr) questionnaire, which was adapted from the method of 3-day 24hdr [20]. Participants were instructed to record all food intake at home and away from home in the previous 2 days (one weekday and one weekend day). Consumptions of condiments were also recorded through a questionnaire. All questionnaires were completed after the patient registering in *Tuberculosis management information system* and before anti-tuberculosis treatment.

Total energy, four macronutrients and sixteen micronutrients were evaluated. Nutrient intakes of each patient were converted to calories, weight of protein and weight of micronutrients based on Chinese Food Composition Tables (CFCT) 2004 [21].

To evaluate whether the patients’ nutrients were sufficient, we compared mean daily nutrient intakes of TB patients to Recommended Nutrient Intakes (RNIs) and Adequate Intakes (AIs) by Chinese dietary reference intakes (DRIs) 2013. Recommended Nutrient Intakes is an estimate of the amount of a nutrient that meets the requirements of most people (97–98%) within a specific physiological group (sex, age, body size, physical activity, type of diet). Adequate Intakes means a recommended intake value based on observed or experimentally determined approximations or estimates of nutrient intake by a group (or groups) of healthy people [16].

### Statistical analysis

Data of daily nutrient intakes were presented as means ± standard deviation (SD). As dietary recommendations are different for men and women, we compared TB patients’ daily nutrient intakes with DRIs by gender. Since protein-calorie malnutrition (PCM) is the most common form of undernutrition in TB patients, we only examined factors related to insufficient energy and protein intakes. Univariate logistic regression analysis was used to identify potential risk factors associated with inadequate energy and protein intakes in 300 TB patients. Age, gender, county and severity of TB were considered to be possible confounders in the multiple logistic regression model with stepwise selection. Patients were classified into two groups: below RNI/AI and above RNI/AI. A *P*-value of less than 0.05 was considered statistically significant. All analyses were performed using SAS 9.4 (SAS Institute Inc., Cary, NC, USA).

### Quality control

The study was carried out on the basis of China Health and Nutrition Survey (CHNS) 2015, from which the investigating method and tool were used in our study. All on-site investigations were carried out by the county-level CDC and interviewers were trained with a standard protocol. Data was checked for completeness and accuracy on the day of investigation and sampled by provincial CDC for verification later. All data was double-entered into a database specially designed for this project.

## Results

### Characteristics of the subjects

All TB patients registered from Nov 1st, 2015 to May 11th, 2017 in Lin county were included in our study, while less than 40% of notified TB patients in Lingyun county have participated in this study.

The majority (53.7%) of TB patients aged between 18 and 49, with the mean age being 45.5 years old (standard deviation (SD):18.7). More than half (68.7%) of them were males. Most TB patients were married, employed and didn’t attend junior middle school and above, with annual household income less than ¥20, 000. Only 39.7% of them consumed alcohol, while 46.7% of them were smokers (Table 1).

**Table 1:** Socio-demographic and behavioral characteristics for 300 TB patients

Characteristics		N	N%
Socio-demographic factors			
Age group	18–49	161	53.7
	50–64	86	28.7
	≥65	53	17.7
	Mean (±SD)	45.5 (±18.7)	
Gender	Male	206	68.7
	Female	94	31.3
Education	Primary school and below	161	53.7
	Junior middle school and above	139	46.3
Marital status	Married	209	69.7
	Other marital status	91	30.3
Occupation	Employed	178	59.3
	Unemployed	122	40.7
Annual household income^a^	< 20,000	136	45.3
	20,000-40,000	79	26.3
	≥40,000	20	6.7
	Refusal or unknown	65	9.7
	Mean (±SD)	18,203.1 (±17,622.2)	
Behavioral factors			
Alcohol consumption	Yes	119	39.7
	No	181	60.3
Smoking status	Smoker	140	46.7
	Non-smoker	160	53.3
Eating out-of-home	Yes	58	19.3
	No	242	80.7

^a^65 patients who refused or with missing data were excluded from this analysis

### Daily energy and nutrient intake

The average daily energy was1655.0 kcal (SD: 619.3 kcal) and 1360.3 kcal (SD: 552.1 kcal) for males and females, respectively, which were significantly lower than recommended daily energy intake by DRIs (i.e., 2250 kcal and 1800 kcal for males and females). The protein intake was 44.6 g (SD: 18.2 g) and 35.9 g (SD: 12.3 g) for male and female TB patients, respectively. The protein daily intakes for our surveyed TB patients was significantly lower than daily protein intakes that were recommended by DRIs, which were 65 g and 55 g for males and females, respectively (Table 2). Our results also indicated that the male patients were more likely subjected to malnutrition. For example, percentages of male patients that had daily intakes below RNI/AI were 87.4 and 90.8% for daily energy intakes and protein intakes, respectively. Percentages for male patients were 59.9 and 58.4% for daily energy and protein intakes, respectively. As for micronutrients, for male TB patients, the mean daily intakes of retinol, niacin, vitamin E, sodium, iron, Manganese, copper and Phosphorus were higher than RNI/AI, while calcium, i.e., 216.3 mg, intake was severely less than RNI/AI (800 mg) (Table 3). For the female patients, the mean daily intakes of vitamin E, copper and sodium were higher than RNI/AI. However, the intakes of riboflavin, potassium and calcium were severely inadequate.

**Table 2:** Daily energy and macronutrient intakes of TB patients compared with DRIs

Energy and macronutrient intakes	Males (*n* = 206)			Females (*n* = 94)		
	RNI/AI	Mean (SD)	Below RNI/AI(%)	RNI/AI	Mean (SD)	Below RNI/AI(%)
Calories (Kcal)	2250	1655.0 (619.3)	180 (87.4)	1800	1360.3 (552.1)	78 (83.0)
Total Protein (g)	65	44.6 (18.2)	187 (90.8)	55	35.9 (12.3)	86 (91.5)
Total Fat (g)	–	73.0 (50.5)	–	–	68.9 (48.3)	–
Total Carbohydrates (g)	–	212.1 (117.2)	–	–	154.8 (58.0)	–
Total fiber (g)	–	7.6 (4.3)	–	–	5.7 (2.6)	–

**Table 3:** Daily micronutrient intakes of TB patients compared with DRIs

Micronutrient intakes	Males (n = 206)			Females (n = 94)		
	RNI/AI	Mean (SD)	Below RNI/AI(%)	RNI/AI	Mean (SD)	Below RNI/AI(%)
Retinol (μg)	800	807.5 (994.0)	135 (65.5)	700	603.9 (626.7)	68 (72.3)
Thiamin (mg)	1.4	0.9 (0.5)	180 (87.4)	1.2	0.7 (0.3)	88 (93.6)
Riboflavin (mg)	1.4	0.6 (0.3)	203 (98.5)	1.2	0.5 (0.2)	94 (100)
Niacin (mg)	12	12.6 (5.3)	94 (45.6)	15	10.3 (4.7)	78 (83.0)
Vitamin E (mg)	14	53.6 (39.6)	13 (6.3)	14	57.1 (40.0)	4 (4.3)
Vitamin C (mg)	100	96.6 (75.3)	130 (63.1)	100	70.2 (55.8)	74 (78.7)
Potassium (mg)	2000	1309.2 (636.7)	180 (87.4)	2000	1011.7 (351.0)	94 (100)
Sodium (mg)	1500	2204.4 (1204.8)	67 (35.5)	1500	2054.5 (1123.4)	28 (29.8)
Calcium (mg)	800	216.3 (126.2)	205 (99.5)	800	176.9 (86.5)	94 (100)
Magnesium (mg)	330	221.0 (112.0)	174 (84.5)	330	166.0 (56.6)	92 (97.9)
Iron (mg)	12	14.2 (6.8)	86 (41.8)	20	10.8 (3.7)	92 (97.9)
Manganese (mg)	4.5	4.7 (2.3)	126 (61.2)	4.5	3.5 (1.3)	83 (88.3)
Zinc (mg)	12.5	5.5 (2.9)	196 (95.2)	7.5	4.1 (1.5)	90 (95.7)
Copper (mg)	0.8	1.2 (1.2)	72 (35.0)	0.8	0.9 (0.3)	53 (56.4)
Phosphorus (mg)	720	745.7 (382.1)	116 (56.3)	720	571.8 (173.0)	74 (78.7)
Selenium (μg)	60	26.3 (15.3)	200 (97.1)	60	20.2 (10.2)	93 (98.9)

### Factors associated with low energy and low protein intakes in 300 TB patients

Our univariate analyses suggested that employment status influenced energy intake, with lower energy consumption was found for the patients that were unemployed (Table 4). Furthermore, annual household income was another factor that affected daily energy intakes, for the patients whose annual salary was more than ¥40,000 tended to have enough energy intakes. In multivariate analysis (Table 5), after adjusting for other factors, unemployed participants were more likely to have low energy intake (OR: 3.542; 95%CI: 1.471, 8.530) (*p* < 0.01). Meanwhile, lower protein intake was associated with eating at home. The possibility of low protein intake for people eating out-of-home was much lower than those eating at home (OR: 0.328; 95% CI: 0.133, 0.809) (*p* < 0.05).

**Table 4:** Factors associated with low energy and low protein intakes using univariate regression analysis in 300 TB patients

Associated factors		Energy		Protein	
		OR (95%CI)	*P*	OR (95%CI)	*P*
Socio-demographic factors					
Age	18–49	1.0		1.0	
	50–64	1.032 (0.498, 2.138)	0.9319	2.039 (0.730, 5.698)	0.1741
	≥65	2.252 (0.746, 6.797)	0.1499	1.542 (0.498, 4.778)	0.4530
Area	Lin county	1.0		1.0	
	Linyun county	0.639 (0.329, 1.240)	0.1856	1.509 (0.676, 3.369)	0.3158
Gender	male	1.0		1.0	
	female	0.704 (0.358, 1.386)	0.3098	1.092 (0.460, 2.593)	0.8415
Severity	mild	1.0		1.0	
	severe	0.851 (0.425, 1.704)	0.6486	0.859 (0.370, 1.911)	0.7224
Education	Primary school and below	1.0		1.0	
	Junior middle school and above	1.503 (0.547, 2.207)	0.8780	0.923 (0.418, 2.037)	0.8429
Marriage	Married	1.0		1.0	
	unmarried	1.708 (0.781, 3.735)	0.1797	0.859 (0.370, 1.991)	0.7224
Occupation	Employed	1.0		1.0	
	Unemployed	3.364 (1.499, 7.550)	0.0033	1.412 (0.612, 3.257)	0.4181
Annual household income	< 20,000	1.0		1.0	
	20,000-40,000	0.766 (0.333, 1.762)	0.5311	0.905 (0.336, 2.438)	0.8437
	≥40,000	0.289 (0.097, 0.866)	0.0266	0.499 (0.126, 1.969)	0.3207
Behaviroral factors					
Alcohol consumption	No	1.0		1.0	
	Yes	1.372 (0.690, 2.731)	0.3670	1.350 (0.585, 3.113)	0.4821
Smoking status	Non-smoker	1.0		1.0	
	Smoker	0.766 (0.399, 1.472)	0.4243	0.482 (0.213, 1.092)	0.0802
Eating out-of-home	No	1.0		1.0	
	Yes	0.628 (0.294, 1.228)	0.2280	0.302 (0.132, 0.693)	0.0047

Note: Intake above RNI/AI was assigned as 0, intake below RNI/AI was assigned as 1. The sample size of analysis of annual household income was 235

**Table 5:** Factors associated with low energy and low protein intakes using multivariate regression analysis in 300 TB patients

Associated factors			OR (95% CI)	*P*
Energy	Age	18–49	1.0	
		50–64	1.010 (0.452, 2.260)	0.9800
		≥65	1.916 (0.560, 6.555)	0.3002
	Gender	Male	1.0	
		female	0.512 (0.246, 1.067)	0.0741
	Area	Lin county	1.0	
		Linyun county	0.631 (0.286, 1.394)	0.2550
	Severity	mild	1.0	
		severe	1.192 (0.546, 2.605)	0.6595
	Occupation	Employed	1.0	
		Unemployed	3.542 (1.471, 8.530)	0.0048
	Annual household income	< 20,000	1.0	
		20,000-40,000	1.242 (0.536, 2.877)	0.6132
		≥40,000	0.366 (0.115, 1.163)	0.0885
Protein	Age	18–49	1.0	
		50–64	1.460 (0.490, 4.353)	0.4927
		≥65	0.985 (0.285, 3.401)	0.9813
	Gender	Male	1.0	
		female	1.133 (0.468, 2.741)	0.7817
	Area	Lin county	1.0	
		Linyun county	1.284 (0.489, 3.370)	0.6122
	Severity	mild	1.0	
		severe	0.690 (0.266, 1.792)	0.4461
	Eating out-of-home	No	1.0	
		Yes	0.328 (0.133, 0.809)	0.0155

Note: Intake above RNI/AI was assigned as 0, intake below RNI/AI was assigned as 1. The sample size of analysis of annual household income was 235

## Discussion

Our study demonstrated that intakes of both macronutrients and selected micronutrients were inadequate for most TB patients, regardless of their gender. Protein-calorie malnutrition (PCM), characterized by inadequate intakes of both protein and total calories [8], was common in TB patients. This finding was consistent with previous studies in China [11–13]. PCM can reduce effectiveness of some components that play important roles in cell-mediated immunity [8].

We found that mean daily intakes of many micronutrients were below RNI/AI, especially calcium. One potential explanation is that TB patients do not consume enough fish, shrimp and dairy products [22], which are all calcium-rich food. Calcium plays an important role in the recovery of TB as the calcification of lesions requires a large amount of calcium, thus calcium deficiency can lead to delayed recovery [12]. Our results suggested that over 95% TB patients didn’t have adequate intakes of essential micronutrients such as riboflavin, zinc and selenium. Riboflavin is associated with the metabolisms of iron and niacin and its deficiency will compromise oxidant defense mechanisms [23]. Zinc has an essential role in vitamin A metabolism and supplementation of zinc will increase immune function in TB patients [24]. Selenium is reported to have an important function in maintaining the immune process and is beneficial for the clearance of mycobacteria [25]. However, sodium consumption was higher than RNI/AI in TB patients. Researches conducted in other populations [26–28] in China also showed that high sodium consumption remained a public health problem. Nevertheless, the quality of diet seemed to be improved, as the mean daily intake of vitamin E was much higher than RNI/AI and the values that were reported in previous studies [11–13].

Furthermore, we discerned that eating out-of-home was related to higher intake of protein. Relevant studies conducted among other populations in China [29–31] also demonstrated that there was an increase in protein intake while eating out-of-home. The reason for this phenomenon may be that people tend to choose food with better tastes and higher nutritional values when they eat away from home [30]. Additionally, eating out-of-home has more frequently been associated with higher socioeconomic status (measured by higher education level and household income) [29, 32, 33], as people in higher socioeconomic status are more likely to afford frequent away-from-home food consumption. We also discovered that the inadequate energy intake was significantly higher among unemployed TB patients, which is consistent with studies conducted in other countries [34, 35]. This is most likely because that the patients with low socioeconomic status may not be able to afford nutritious food.

Malnutrition can also have a negative impact on the treatment of TB. Malnutrition will reduce the level of protein in the patient’s body, and thus delayed the recovery of lesions. This is probably because sufficient protein not only can promote the recovery of lesions during the treatment, but also can increase the number of anti-tuberculosis drug carriers in the treatment, therefore, concentration of anti-tuberculosis drugs in the blood will rise, promoting sputum conversion [36]. A study in China showed that 56% of patients’ sputum culture were negative after 20 days of treatment in the nutritional support group, which was significantly higher than 28% in the general treatment group [37].

### Strengths and limitations

Our study initiated in examining factors associated with TB patients’ nutrient intake and was also the first study in China to compare nutrient intakes of TB patients with the latest Chinese Dietary Reference Intakes (DRIs) 2013. However, there are still some limitations in our study. Firstly, the 24hdr method has its own shortcomings. Reliance on the memory of participants and recall bias could result in an underestimation of nutrient intakes [38]. Secondly, considering that more times we interviewed, less cooperate would TB patients be because they may be worried about being discriminated against, so we took the 2-day 24hdrs method instead of the commonly used 3-day 24hdrs, but the reliability may decrease. A study conducted among African American youth showed that reliability estimates for 3-day 24hdrs ranged from 27 to 62%, while, for 2-day 24hdrs, it ranged from 19 to 52% [39]. And, when measuring the consumption of condiments, we didn’t take the household weighing method commonly used in nutrition survey. Thirdly, we hope to enroll all eligible TB patients in these two counties notified from Nov 1st, 2015 to May 11th, 2017. All notified TB patients in Lin county were included in our study, while TB patients enrolled in our study accounted for less than 40% of all notified TB patients in Lingyun county during that period. Moreover, we measured socioeconomic status of TB patients only by annual household income and this was not enough to capture the economic disparities. All of these may result in that our findings could not be generalized to all the local people.

## Conclusions

In this study, we showed that in poor areas of China, the intakes of macronutrients and most micronutrients for TB patients were lower than DRIs, especially for those unemployed and eating at home. To reach effective treatments for TB patients, our study calls for promotion of awareness of significance of nutritional support in treatment process. In some cases, interventions in TB patients’ nutritional intakes are also required. Most importantly, the government should provide financial support for the patient so that they can afford enough food supplies.

## Data Availability

The datasets used or analyzed during the current study are available from the corresponding author on reasonable request.

## Abbreviations

24hdr: 24-h dietary recall
AI: Adequate Intake
BMR: basal metabolic rate
CDC: Center for disease control and prevention
CFCT: Chinese Food Composition Tables
CHNS: China Health and Nutrition Survey
DRIs: Dietary Recommended Intakes
PCM: Protein-calorie malnutrition
RNI: Recommend Nutrient Intake
SD: Standard deviation
TB: tuberculosis
WHO: World Health Organization
